# Venous Thrombosis in Multiple Sclerosis Patients after High-Dose Intravenous Methylprednisolone: The Preventive Effect of Enoxaparin

**DOI:** 10.1155/2011/785459

**Published:** 2011-12-25

**Authors:** Hossein Kalanie, Ali Amini Harandi, Shapoor Alidaei, Daryoosh Heidari, Saeed Shahbeigi, Mehdi Ghorbani

**Affiliations:** ^1^Mehr Hospital, Shahid Beheshti University of Medical Sciences, Tehran 19857-17443, Iran; ^2^Loghman Hospital, Shahid Beheshti University of Medical Sciences, Tehran 19857-17443, Iran; ^3^Mehr Hospital, Shahid Beheshti University of Medical Sciences, Tehran 19857-17443, Iran; ^4^Department of Cardiology, Faculty of Medicine Damascus University, P.O. Box 8487, Damascus, Syria

## Abstract

*Aim*. This study was designed to examine the possible role of high-dose intravenous methylprednisolone (IVMP) in the development of venous thrombosis (VT). The cerebral one anecdotally had been reported in patients with relapsing remitting multiple sclerosis (RRMS) in acute attacks and the possible preventive role of enoxaparin. *Material and Methods*. From a pool of 520 patients, 388 patients with definite RRMS who fulfilled entry characteristics were selected and randomly received either a 5-day course of daily 1 gr IVMP or the aforementioned plus 5 days of daily subcutaneous 40 units of enoxaparin according to a predefined protocol. *Results*. Mean age, gender ratio, mean relapse rate, and EDSS were similar in both groups of patients (*P* > 0.05). Finally, 366 patients remained in the study. Of 188 patients treated with IVMP with 855 relapses, 5 developed VT (0.37% per patient per year and 0.58% per each course of IVMP) within 3 to 15 days of starting therapy. None of the 178 patients who experienced 809 relapses who received IVMP plus enoxaparin developed such complications. *Conclusion*. The study implies that high-dose IVMP in MS exacerbation may increase the risk of VT and prophylactic anticoagulant treatment in this setting is warranted.

## 1. Introduction

Intravenous corticosteroid (CS) is a well-defined treatment for multiple sclerosis (MS) relapses and high doses of methylprednisolone (MP) (bolus of 500–1000 mg of MP daily for 3 to 5 days followed or not by oral prednisolone), routinely delivered in many neurological centers with good tolerance [1, 2]. Anecdotal report linked high-dose CS treatment in MS to an increased risk of developing cerebral venous thrombosis (CVT) [3, 4]. We also observed now and then MS patients in relapses who developed deep vein thrombosis (DVT) in their lower extremities during perfusion of high-dose MP. Because high doses of intravenous methylprednisolone (IVMP) are routinely delivered in a wide variety of other neurological and medical disorders, such an association may have an impact on prophylactic strategies against venous thromboembolism during such a treatment. In this study, we tried to examine the possible role of high-dose IVMP in the development of venous thrombosis in RRMS patients in acute relapse and evaluate the possible preventive effect of daily subcutaneous enoxaparin (low-molecular-weight heparin) on this life threatening side effect.

## 2. Material and Method

The study took place at Mehr Hospital, Tehran, Iran. The study period was from March 2002 to April 2009. Searching the clinical files of 520 Caucasian Iranian patients attending our MS clinic [5] with clinically definite MS, according to the criteria of McDonald [6], 380 cases of relapsing-remitting type (RRMS) who fulfilled the inclusion criteria were selected. These criteria consisted of an age range between 14 and 45 years, a Kurtzke Expanded Disability Status Score (EDSS) [7] not over 4. Exclusion criteria were including overt diabetes or obesity, CS therapy or contraceptive use for at least 12 months, history of previous venous thrombosis or cigarette smoking, positive pregnancy test, abnormal haemostatic parameters (serum fibrinogen, antithrombin III, C and S protein, absence of resistance to activated C protein due to factor V leiden mutation), and positive Anticardiolipin (IgG and IgM) antibodies. Also all patients with other clinical conditions that could potentially cause the neurological clinical and/or other laboratory findings were excluded (e.g., Lupus erythematosus, Behcet's disease, Sjogren disease, vasculitis, HIV infection, syphilis, human T-cell leukemia/lymphoma virus (HTL V 1 and 2), and cerebral autosomal dominant arteriopathy with subcortical infarcts and leukoencephalopathy). Written informed consent was obtained from all patients before participating in study. Patients then were assigned to a serial list of random numbers. Odds were assigned to receive a 5-day course of daily 1 gr IVMP following an exacerbation event. Evens received the above-mentioned drug plus daily 40 mg subcutaneous enoxaparin.

Gastroduodenal protection was achieved by using 20 mg of omeprazole capsule daily during the study period. Patients were advised to call the hospital in the event of any neurological symptoms or disability. In such cases, the assessing physician examined the patient to confirm a possible relapse and if so admitted the patient to the hospital to receive the above-mentioned therapy. An MS exacerbation was defined as either the onset of new symptoms and signs or deterioration in the existing symptoms and signs of at least 24 hours in duration without concomitant fever, accompanied by documented change in the neurological examination. A change of 1.0 or greater in the EDSS grading was considered significant [8]. Upon admission and before beginning therapy, all patients had a chest X-ray, complete blood count, electrolytes, BUN, serum: creatinine, aspartate aminotransferase (SGOT), alanine aminotransferase (SGPT), alkaline phosphatase, bilirubin, glucose, urinalysis, intermediate strength purified protein derivative skin test for tuberculosis, and pregnancy test if appropriate [9]. Patients were advised to be up and about between infusions to reduce the possible effect of immobility. Patients were monitored before and during the treatment course and were reviewed every week for the first 4 weeks and then every 3 months as a routine followup. If there was any sign referable to DVT such as pain or swelling of legs or CNS involvement such as headaches, vomiting, focal signs, they underwent duplex sonography or brain MR venography, respectively. Paired-sample *t*-test and independent sample *t*-test were statistical methods used.

## 3. Results

Of 380 patients having clinically definite RRMS according to McDonald's criteria, 14 dropped out of the study not being able to follow the protocol (2 had plan for pregnancy, 4 started on contraceptive pills, and 6 missed their followup), 188 were treated with IVMP, and 178 treated with IVMP plus subcutaneous enoxaparin following an exacerbation event. Entry characteristics of patients are summarized in Table 1.

Mean age, gender ration, mean annual relapse rate, and EDSS were similar in both groups of patients (*P* > 0.05). Of 188 patients who received only IVMP (130 female and 58 male) with a total of 855 relapses, 5 developed venous thrombosis (0.37% per patient per year and 0.58% per each course of IVMP).

Two females aged 32, 37 and one male aged 39 had DVT involving lower extremities; 2 happened at day 7 and 10 in the first course, 1 at day 10 in the second course, respectively, and another 2 females aged 35 and 39 developed CVT involving the left sigmoid and sagittal sinuses, respectively, at days 5 and 15 during the first and fifth course of therapy (Table 2).

Of 178 patients who received IVMP plus subcutaneous enoxaparin (124 females and 54 males) with 809 relapses, none had signs of VT.

Using Fisher's Exact Test to compare thrombotic event between two groups, a *P* value of 0.061 was obtained (5 thrombotic patients in 188 of MS patients) and 0 (0 thrombotic patients in 178 of MS patients, *P* = 0.061).

## 4. Discussion

Both DVT and CVT have some known predisposing and etiologic factors. For DVT, they are reduced mobility, obesity, malignant disease, history of venous thromboembolism, thombophilic disorders, and older age [10]. CVT although a rare condition induced by multiple etiologies including infectious disease, inflammatory diseases (in some cases anticoagulants and CS are associated in their treatment), puerperium and pregnancy, brain tumor and trauma, coagulation abnormalities, neoplasic disease, and drugs [11, 12]. Despite the diversity of cause, the etiology of cerebral venous thrombosis remains undetermined in 20–30% of the cases [13, 14]. Drugs can play as a predisposing factor in both conditions. One of the medications which have been under suspicion in this respect, especially CVT, is the use of high-dose IVMP in treating MS patients in acute relapse. In a cohort of 120 consecutive patients with acute CVT, diagnosed either by digital subtraction angiography or magnetic resonance imaging and MR angiography, 4 patients who developed CVT during intravenous CS treatment (≥500 mg/day over 5 days) for a relapse of MS were identified along with 2 more cases who received high-dose CS for treatment of optic neuritis [15]. Overall, this amounts to 5% of patients who developed CVT during CS treatment, a rate as high as could be expected in protein C or S deficiency [16]. A causative link also has been proposed between lumbar puncture (LP) and CVT but it was not always the case [17]. In most instances, LP played its role whenever other predisposing factors such as high-dose CS use is in action [13, 14, 18, 19].

High-dose IVMP for 3–5 days is frequently used for rapid and strong immunosuppression in acute autoimmune disease and in transplantation medicine. Glucocorticoids are commonly thought to be procoagulant increasing the risk of thromboembolic complications like DVT and pulmonary embolism [20–22]. The acute femoral head osteonecrosis occurring during or after high-dose steroid treatment also may be explained by hypercoagulopathic mechanisem [23]. Cushing syndrome features high-glucocorticoid secretion and associated hypercoagulable state often involves an increase in Von Willebrand factors [24]. Glucocorticoids usage especially at high doses is complicated by adverse outcome such as thrombotic events in condition like myeloma and osteoporosis [25]. However, hypercoagulopathy induced by high-dose IVMP is a controversial issue. In one study investigating the effect of high-dose IVMP on sensitive markers of coagulation and fibrinolysis activation, authors did not find evidence for a pronounced acute prothrombotic state induced by this medication [26] and pointed the role of underlying disease for an elevated thrombolytic risk. Anecdotal reports in recent years may suggest that MS per se may provide a suitable background for VT [17, 24, 27].

In this study, we tried to examine the possible role of high-dose IVMP in the genesis of venous thrombosis while excluding almost all known possible predisposing factors including poor mobility during treatment of acute relapses in RRMS. This study implies that VT can occur with an estimated incidence of 0.58% during any course of IVMP while treating an MS relapses even in the absence of other risk factors and prophylactic enoxaparin can prevent this side effect.

Interestingly enough 4 out of our 5 patients are above 35 years of age and 4 of them are female which may denote the role of age and gender as predisposing factors in our patients. In fact, estrogens have many different effects on the coagulation system. These include increases in the level of procoagulant factors VII, X, XII, and XIII and reductions in the anticoagulant factors protein S and antithrombin [28].

The study was done on small scale and if we had 280 subjects in each group, on the basis of power statistical calculation with *β* = 20%, we would have a *P* value of 0.05 which is a warning. Taken together, we believe that there is a synergistic effect between high-dose IVMP and MS. In fact, when IVMP acts on MS immunopathology, they may then provide a suitable background for occurrence of VT. In this setting LP by its own mechanism [29] may have a compounding effect. Since we tried to discard susceptible patients for thrombosis, the final rate of this side effect might have been much higher in general population of MS therefore prophylactic treatment with enoxaparin may be warranted in these patients.

We would like to emphasize that our study is done on a limited number population with its own specific genetic, environmental, and cultural background [30], and perhaps the results cannot be extended with certainty to other MS populations of other nations. Therefore, similar studies in a larger scale on other ethnic population on different continents are warranted and if it confirms our hypothesis, it could to save life of many patients by a reasonable prophylactic therapy. In that case, whatever the cost effectiveness may be, the high mortality rate of CVT and the danger of pulmonary emboli from DVT will make it negligible.

## Figures and Tables

**Table 1:** Entry characteristics (mean ± SD) of patients.

	*IVMP	**IVMP + SCE
Patients	188	178
Age	29.4 ± 7.1	29.8 ± 7.2
Sex	2.2 : 1	2.2 : 1
Gender Ratio F : M	2.2 : 1	2.2 : 1
Mean Annual Relapse Rate	0.65	0.65
EDSS	3.4 ± 0.50	3.4 ± 0.82

*Intravenous methylprednisolone.

**IVMP + subcutaneous enoxaparin.

EDSS: the Expanded Disability Status Scale.

**Table 2:** Characteristic of patients on high-dose IVMP with venous thrombosis.

	Sex	Age	Course number	Day of diagnosis	Involved site	Type of thrombosis
1	F	32	1	7	Left leg	*DVT
2	F	37	1	10	Left leg	DVT
3	M	39	2	10	Both legs	DVT
4	F	35	1	5	Left sigmoid	**CVT
5	F	39	5	15	Sagittal Sinus	CVT

*DVT: deep vein thrombosis.

**CVT: cerebral vein thrombosis.
